# The Potential of Pre-fermented Juice or *Lactobacillus* Inoculants to Improve the Fermentation Quality of Mixed Silage of Agro-Residue and Lucerne

**DOI:** 10.3389/fmicb.2022.858546

**Published:** 2022-04-28

**Authors:** Lin Mu, Qinglan Wang, Xin Cao, Hui Li, Zhifei Zhang

**Affiliations:** Department of Grassland Science, College of Agronomy, Hunan Agricultural University, Changsha, China

**Keywords:** pre-fermented juice, bacterial inoculants, microbial community, agro-residue, mixed silage

## Abstract

The objective of this study was to determine the effect of pre-fermented juice, *Lactobacillus plantarum*, and *L*. *buchneri* on chemical composition, fermentation, aerobic stability, dynamics of microbial community, and metabolic pathway of a mixture of lucerne, wheat bran (WB), and rice straw (RS). All mixtures were ensiled for 1, 3, 5, 7, 15, 30, and 45 days after treatment with uninoculated (control, C); *L*. *plantarum* [LP, 1 × 10^6^ cfu/g of fresh weight (FW)]; *L. buchneri* (LB, 1 × 10^6^ cfu/g of FW); LP + LB (LPB, 1 × 10^6^ cfu/g of FW of each inoculant); and pre-fermented juice (J; 2 × 10^6^ cfu/g of FW). Four lactic acid bacteria (LAB) species from three genera were cultured from the pre-fermented juice, with *W. cibaria* being dominant. The inoculants increased lactic acid (LA), decreased pH and ammonia nitrogen (AN) compared to C silage at earlier stages of ensiling, and high dry matter (DM) and water-soluble carbohydrate (WSC) content in inoculated silages. Adding LPB increased the abundance of *L. plantarum*, *L. paralimentarius*, and *L. nodensis*, resulting in the lowest pH. Pre-fermented juice enriched *W. cibaria*, *L. sakei*, *L. parabrevis*, *Pseudomonas putida*, and *Stenotrophomonas maltophilia*, mainly enhanced accumulation of acetic acid (AA) and LA, and decreased pH, crude protein losses, AN, and hemicellulose contents. *L. buchneri* and *L. brevis* had a high abundance in LB-treated and J silages, respectively, inhibited undesirable bacteria, and improved aerobic stability with more than 16 days. In addition, the metabolic pathways changed with time and *L. buchneri* inoculants promoted global metabolism. In conclusion, inoculations altered bacterial succession and metabolic pathways in silage; LB and pre-fermented juice enhanced ensiling by promoting pH reductions, enhancing concentrations of LA and AA, and extending aerobic stability more than 16 days.

## Introduction

With livestock production and cultivation of lucerne both increasing in southern China, ensiled lucerne is becoming a common forage source. However, the humid and rainy climate makes it difficult to obtain optimal dry matter (DM) for silage. Fortunately, crop by-product, an important biomass resource in green, sustainable, and intensive agricultural production, is a promising adsorbent to reduce silage moisture. Ensiling lucerne with crop by-products would not only reduce moisture, but also reduce competition between humans and animals for the same food sources (Chen, 2016; Du et al., 2021a). Rice straw (RS) is an abundant agro-residue biomass in China, with most of it incinerated in fields, thereby precluding any use and causing environmental pollution. In addition, wheat bran (WB), a common byproduct of wheat production with abundant carbohydrates and high DM, is widely available for use as an animal feed (Chen, 2016). The addition of WB to paper mulberry silage enhanced fermentation quality and enabled *Lactobacillus plantarum* (LP) to become the dominant microbe. Furthermore, WB can also be combined with RS to produce high-nutrient silage by increasing DM recovery (Jian-Xin et al., 2001; Du et al., 2021b). Therefore, it is believed that mixing WB and RS in lucerne silage could effectively use large quantities of crop by-products and alleviate the feed shortage in tropical areas. However, adding RS and WB in lucerne silage absorbs moisture and increases DM, but concurrently decreases concentrations of lactic acid bacteria (LAB), as these products have a paucity of epiphytic LAB (Mu et al., 2020; Du et al., 2021a).

Various inoculants are used to promote fermentation, namely acid production rate and aerobic stability. The most common inoculants for silage are *L. plantarum* and *L. buchneri* to decrease pH and inhibit yeasts and molds (Guo et al., 2018). In addition, pre-fermented juice, cultured epiphytic microorganisms of crop materials, has been recommended to enhance silage fermentation, as it can be produced at low cost, is environmentally friendly, and an excellent biological source of LAB (Ohshima, 1997). Inoculating pre-fermented juice to alfalfa silage improved the quality of fermentation by decreasing pH through lactobacilli-driven LA production (Wang et al., 2009). Although many researchers have used LAB and pre-fermented juice as inoculants for silage production, results have varied, as microbial content of pre-fermented juice is usually complex and inconsistent (Adesogan, 2008). There are limited studies on the bacterial community in pre-fermented juice, and how pre-fermented juice affects bacterial community succession in lucerne-based silage is unclear. Thus, the objective was to determine whether pre-fermented juice and inoculations affected fermentation quality and microbiome, and microbial taxa of pre-fermented juice that influenced silage fermentation. We hypothesized that pre-fermented juice is constituted by complicated bacteria, which could successively play a role during the ensiling process.

## Materials and Methods

### Silage Preparation

Both lucerne and rice were grown by Chang De, China (longitude 112°06′58″, latitude 29°06′27″, altitude 30 m), whereas WB was from Kangda Agricultural Products Co., Ltd. (Anhui, China). In May 2019, the second crop of lucerne at the 10% bloom stage was harvested (hand clippers) from three plots. The RS was procured after rice was harvested. Lucerne and RS were processed in a fodder cutter and reduced to an overall length of 1–2 cm. Lucerne, WB, and RS were combined in a ratio of 80:15:5, based on preliminary experiments. Additives used were commercial *L. plantarum* and *L. buchneri* inoculants (Yaxin Biotechnology Co., Ltd., Taiwan, China), and pre-fermented juice. The latter was prepared from lucerne that was chopped from three plots and used as replicates. For this, 200 g of chopped forage were mixed with 1 L of distilled water and macerated for 2 min in a blender, filtered (two layers of cheesecloth), and glucose (2 g/100 ml) was added, the solution was well mixed and placed in a sealed plastic container in an incubator at 30°C for 48 h (Tao et al., 2018).

A total of 158 silage mixtures (4 kg lucerne, 0.75 kg WB, and 0.25 kg RS) were prepared, with 105 randomly selected (5 treatments × 7 sampling days × 3 replicates/treatment), as follows: (1) uninoculated (control, C); (2) *L. plantarum* (LP; 1 × 10^6^ cfu/g FW); (3) *L. buchneri* (LB; 1 × 10^6^ cfu/g FW); (4) LP + LB (LPB; 1 × 10^6^ cfu/g FW of each inoculant); (5) pre-fermented juice (J; 2 × 10^6^ cfu/g FW, with 7.61 log cfu/ml and pH 4.16). Inoculants were added to deionized water, and with constant mixing, 20 ml/kg was sprayed as a fine mist on the material to be ensiled. Similarly, 20 ml/kg of fermented juice or distilled water (J and C treatments, respectively), were also applied. All samples (600 g of raw material) were ensiled in vacuum-heat sealed nylon-polyethylene standard barrier bags (200 × 300 mm; Huaguan Printing Co., Ltd., Zhejiang, China). Air was withdrawn from silos; they were sealed with a vacuum extractor (Dafeng Machinery Co., Ltd., Zhejiang, China) and kept at ∼30°C. On Days 1, 3, 5, 7, 15, 30, and 45, triplicate samples were collected to assess fermentation and composition. Assessment of the microbial community was done at Day 5, 15, and 45 of ensiling and for the fermented juice, after 48 h of incubation.

### Chemical Composition and Fermentation Characteristics

A sample of silage (20 g) was put into 180 ml distilled water and processed in a blender for 1 min. The solution was filtered through 2 layers of cheesecloth and pH measured (SI400 pH meter, Spectrum, Aurora, IL). The filtrate was centrifuged (10,000 × g, 15 min, 4°C) and the supernatant was assessed for the following: volatile fatty acids (VFAs), with gas chromatography (GC7890A, Agilent Technologies, Santa Clara, CA), as described by Mu et al. (2020); lactic acid (LA), *via* an Agilent 1260 HPLC system with a UV detector and Acclaim TM organic acid column (Dionex Co., Ltd., Sunnyvale, CA) with 50 mmol/L NaH_2_PO_4_, 0.6 ml/min at 30°C; and ammonia nitrogen (AN) (Broderick and Kang, 1980). Samples were dried at 65°C for 48 h in a forced-draft for DM analysis, ground in a knife mill with a 1-mm screen, and assessed for neutral and acid detergent fiber (NDF and ADF, respectively) (Van Soest et al., 1991), crude protein (CP) (Mu et al., 2020), and water-soluble carbohydrate (WSC) (Mu et al., 2020), with ADF subtracted from NDF to calculate hemi-cellulose (HC) content. The LAB in pre-fermented juice was cultured on MRS agar plates and enumerated (Cai et al., 1998).

### Sequence Analyses of Bacterial Communities

Pre-fermented juice samples (50 ml) were centrifuged at 10,000 × g for 5 min, the supernatant removed, and the residue stored at −80°C. The DNA was isolated (DNA kit, DP812, Tiangen, Beijing, China) according to the manufacturer’s instructions, from frozen-thawed samples of pre-fermented juice and silage. The DNA was quantified with a NanoDrop 2000 and quality determined with 1% agarose gels. Single-molecule real-time (SMRT) sequencing was done, with primers 27F and 1492R to detect 16S rRNA genes, and polymerase chain reaction (PCR) was done as described by Mu et al. (2021). All DNA assessments were done by Biomarker Technologies Corporation (Beijing, China). A PacBio Sequel (Pacific Biosciences, Menlo Park, CA) was used for analyses, with sequences determined as described by Mu et al. (2021). Alpha diversity used Shannon, Simpson’s diversity, Chao1 and rarefaction estimators, principle component analysis (PCA), R heatmaps were prepared as described by Mu et al. (2020), and Venn diagrams (VennDiagram, Version 1.6.16) were used to compare bacterial communities. Microbial functions were determined based on the Kyoto Encyclopedia of Genes and Genomes (KEGG) database using Phylogenetic Investigation of Communities by Reconstruction of Unobserved States (PICRUSt). The BMK Cloud Platform^1^ was used for data analysis.

### Aerobic Stability

To determine aerobic stability, 90 piles of silage were prepared as described for silage, and 60 were randomly chosen and ensiled. After 45 days, bales were opened, mixed thoroughly, and loosely packed into 2-L sterile plastic boxes that were covered with two layers of gauze and held at 30–35°C. Every 2 h, temperatures of air and silage (middle of the bottle) were measured (Smowo MDL-1048A, Tianhe Automation Instrument Co., Ltd., Shanghai, China). Aerobic stability was defined as the interval for silage to become at least 2°C warmer than air. Effects of aerobic exposure on pH were determined after Day 0, 4, 8, 12, and 16 (three boxes per treatment).

### Statistical Analyses

This experiment was conducted as a completely randomized design (5 treatments, with 7 durations of ensiling to assess fermentation and chemical compositions, and 5 durations of aerobic exposure). The GLM procedure (SAS 9.3, SAS Institute Inc., Cary, NC) was used to conduct two-way ANOVA, with fixed effects of treatment, ensiling day, and their interaction. Fixed effects of LP, LB, LPB, and pre-fermented juice were used to assess sequence, bacterial diversity, and succession of inoculations. For all analyses, *P* < 0.05 was considered significant and differences were located with Tukey’s.

## Results

### The Chemical Composition of Fresh Forage

Contents of NDF, ADF, and DM were higher (*P* < 0.05) in RS than lucerne, whereas the CP content was lower (*P* < 0.05) in RS than lucerne. The WB had higher (*P* < 0.05) DM and HC contents, but lower (*P* < 0.05) NDF, ADF, and CP content than lucerne. However, the mixture had lower (*P* < 0.05) NDF, ADF, and CP and higher (*P* < 0.05) DM and HC compared to lucerne (Table 2).

**TABLE 1:** Mean (SEM^1^) chemical composition before ensiling (n = 3 samples).

Item^2^	AF	RS	WB	F
DM (g/kg FW)	163 ± 4.63^C^	924 ± 0.33^A^	913 ± 1.73^A^	364 ± 4.62^B^
Crude protein (g/kg DM)	219.83 ± 0.01^A^	30.16 ± 0.25^D^	167.01 ± 0.17^C^	172.37 ± 0.22^B^
NDF (g/kg DM)	522.92 ± 1.29^B^	680.16 ± 1.46^A^	459.43 ± 3.34^D^	489.05 ± 3.45^C^
ADF (g/kg DM)	389.26 ± 5.75^B^	538.14 ± 0.63^A^	224.90 ± 2.89^D^	321.65 ± 5.83^C^
Hemicellulose (g/kg DM)	139.76 ± 3.52^C^	142.02 ± 0.83^C^	239.92 ± 3.11^A^	167.41 ± 3.02^B^
WSC (g/kg DM)	78.03 ± 0.98^A^	34.51 ± 1.66^B^	83.79 ± 0.89^A^	79.85 ± 2.27^A^

*^A–D^Within a row, means without a common superscript differed (P < 0.05).*

*^1^SEM, standard error of the mean.*

*^2^DM, dry matter; FW, fresh weight; WSC, water-soluble carbohydrates; NDF, neutral detergent fiber; ADF, acid detergent fiber. FW, fresh weight; DM, dry matter. AF, fresh lucerne; RS, rice straw; WB; wheat bran; F, mixture.*

**TABLE 2:** Effects of additives on fermentative characteristics of silages.

Item	Treatment^1^	Day of ensiling							SEM^2^	*P*-value^3^		
		1	3	5	7	15	30	45		T	D	T × D
pH	C	4.93^A^	4.52^AB^	4.23^A^	4.20^A^	4.20^A^	4.16^AB^	4.17^A^	0.04	<0.001	<0.001	<0.001
	J	4.93^A^	4.43^B^	4.19^BC^	4.17^B^	4.20^A^	4.17^AB^	4.15^BC^				
	LB	4.96^A^	4.58^A^	4.20^BC^	4.14^C^	4.19^AB^	4.18^A^	4.17^A^				
	LP	4.91^A^	4.49^AB^	4.20^B^	4.18^B^	4.19^A^	4.17^AB^	4.16^AB^				
	LPB	4.96^A^	4.48^AB^	4.18^C^	4.18^B^	4.17^B^	4.15^B^	4.14^C^				
Lactic acid	C	8.29^B^	19.20^B^	30.34^B^	35.46^AB^	41.51^AB^	41.86^A^	40.16^A^	1.49	<0.001	<0.001	<0.001
(g/kg DM)	J	9.74^A^	26.84^A^	35.02^A^	37.40^A^	39.90^C^	40.26^B^	40.07^A^				
	LB	10.39^A^	16.13^C^	30.93^AB^	35.35^B^	40.86^AB^	41.17^AB^	39.08^B^				
	LP	10.40^A^	19.23^B^	32.01^AB^	35.09^B^	40.75^BC^	41.24^AB^	39.80^A^				
	LPB	10.01^A^	20.41^B^	30.76^B^	35.59^AB^	41.71^A^	42.16^A^	39.77^A^				
Acetic acid	C	4.39^A^	6.19^C^	9.38^A^	9.81^B^	11.74^AB^	12.35^A^	11.72^B^	0.57	<0.001	<0.001	<0.001
(g/kg DM)	J	4.26^AB^	8.55^A^	9.31^A^	9.63^BC^	12.01^A^	14.08^A^	12.77^A^				
	LB	4.09^B^	6.47^C^	6.65^C^	10.08^A^	11.63^AB^	12.73^A^	12.73^A^				
	LP	4.26^AB^	7.77^B^	9.26^A^	9.61^C^	11.24^B^	12.50^A^	12.77^A^				
	LPB	4.18 ^AB^	4.22^D^	8.76^B^	10.10^A^	11.16^B^	13.21^A^	12.01^B^				
Propionic acid	C	0.15^AB^	0.17^C^	0.32^A^	0.52^A^	0.71^A^	0.76^A^	0.86^A^	0.10	<0.001	<0.001	<0.001
(g/kg DM)	J	0.14^B^	0.30^A^	0.34^A^	0.42^A^	0.68^A^	0.78^A^	0.70^B^				
	LB	0.15^A^	0.16^D^	0.16^B^	0.42^A^	0.51^A^	0.33^B^	0.79^A^				
	LP	0.15^AB^	0.24^B^	0.32^A^	0.40^A^	0.69^A^	0.31^B^	0.86^A^				
	LPB	0.16^A^	0.15*^E^*	0.30^A^	0.54^A^	0.57^A^	0.75^A^	0.66^B^				
Butyric acid	C	0.06^B^	0.06^A^	0.06^C^	0.11^BC^	0.11^C^	0.11^A^	0.12^A^	0.00	<0.001	<0.001	<0.001
(g/kg DM)	J	0.06^B^	0.06^B^	0.11^A^	0.11^BC^	0.12^A^	0.11^A^	0.12^A^				
	LB	0.06^A^	0.06^B^	0.06^BC^	0.11^B^	0.11^AB^	0.10^A^	0.11^BC^				
	LP	0.06^B^	0.06^B^	0.06^B^	0.11^C^	0.11^AB^	0.11^A^	0.11^AB^				
	LPB	0.06^B^	0.06^B^	0.06^C^	0.11^A^	0.11^BC^	0.11^A^	0.10^C^				
Ammonia	C	22.58^A^	35.35^A^	38.14^A^	49.21^A^	70.25^A^	74.59^B^	95.13^B^	2.68	<0.001	<0.001	<0.001
nitrogen	J	15.83^B^	33.68^AB^	36.88^A^	42.78^BC^	66.30^A^	79.81^AB^	88.30^C^				
(g/kg TN)	LB	15.12^B^	36.42^A^	38.18^A^	46.90^AB^	66.63^A^	82.27^AB^	101.90^A^				
	LP	16.73^B^	33.50^AB^	36.43^A^	38.98^C^	67.51^A^	78.82^AB^	92.86^BC^				
	LPB	15.30^B^	29.83^B^	37.36^A^	42.77^BC^	67.69^A^	88.32^A^	99.07^AB^				

*^A–D^Within a day of ensiling, groups without a common superscript differed (P < 0.05).*

*^1^C,control; J, pre-fermented juice-treated silage; LP, L. plantarum-treated silage; LB, L. buchneri-treated silage; LPB, L. plantarum + L. buchneri-treated silage.*

*^2^SEM,standard error of mean.*

*^3^treatments; D, ensiling days; T × D, interaction between treatments and ensiling days.*

### Fermentation Products

There were effects of treatment group and ensiling days (*P* < 0.01) on pH, LA, AA, propionic acid (PA), butyric acid (BA), and AN, and there was an interaction between group and days for silage pH, LA, AA, propionic acid (PA), butyric acid (BA), and AN (Table 2). Across groups, pH decreased as ensiling progressed, with all groups having pH < 4.2 at 45 days. On Day 3 and 5, the pH in all inoculants was lower than in C silage. The pH of silage treated with pre-fermented juice had decreased (*P* < 0.05) after 3 days of ensiling and was lower than C silage at Day 45. After 15 days of ensiling, pH of LPB silage was always lower (*P* < 0.05) than in the other four groups.

There was rapid LA production in silage treated with pre-fermented juice, LP, LB, or LPB after 1 day of ensiling. The J silage had the highest (*P* < 0.05) LA concentration among all silages during the initial 7 days of ensiling, whereas the lower LA (*P* < 0.05) concentration in J silage than in C occurred at Day 15 and 30 of ensiling. The LA concentration of LB silage increased most slowly during 3 days of ensiling, and was significantly lower than all other groups at 45 days of ensiling. However, inoculated treatments with pre-fermented juice, LP, LB, or LPB had greater AA concentrations than the uninoculated C group at 45 days of ensiling. Concentration of AA was highest (*P* < 0.05) in J silage at both 3 and 15 days of ensiling. Furthermore, in LB and LPB silages, AA had the slowest (*P* < 0.05) increase in the first 5 days of ensiling, but AA in these two groups generally exceeded that in C silage until the end of the trial. The PA concentration was higher (*P* < 0.05) in J silage than LB silage only was observed after Day 5 of ensiling, and then it became lower (*P* < 0.05) than LB silage. Both LB and LPB silages had lower (*P* < 0.05) BA concentrations than other treatments after 45 days of ensiling. All inoculants reduced (*P* < 0.05) AN concentration relative to C silage after 1 day of ensiling. The addition of pre-fermented juice lowered AN concentration as compared to C silage over the entire ensiling, with LB silage having the opposite trend.

### Chemical Composition

There were effects of group and time (*P* < 0.01) on DM, WSC, CP, NDF, and HC and an interaction for DM, WSC, CP, NDF, and HC. The C silage had lower DM after 45 days of ensiling and tended to have lower WSC contents than other treatments after 7 days of ensiling. The content of WSC in J silage was lowest in the first 5 days of ensiling, with J silage having higher CP and lower HC contents than other treatments after 45 days of ensiling. The contents of NDF and HC decreased markedly in all groups at 45 days of ensiling with no differences in NDF and ADF content in inoculant treatments vs. control after 45 days of ensiling (Table 3).

**TABLE 3:** Effects of additives on chemical compositions of silages.

Item^1^	Treatment^2^	Day of ensiling							SEM^3^	*P*-value^4^		
		1	3	5	7	15	30	45		T	D	T × D
DM	C	357^A^	352^AB^	345^B^	346^C^	347^B^	346^A^	344^B^	0.80	<0.001	<0.001	<0.001
(g/kg FM)	J	361^A^	356^AB^	354^A^	347^BC^	348^B^	350^A^	348^AB^				
	LB	368^A^	349^B^	352^A^	347^B^	348^B^	347^A^	349^A^				
	LP	361^A^	350^B^	352^A^	352^AB^	345^B^	347^A^	348^A^				
	LPB	360^A^	362^A^	354^A^	358^A^	358^A^	348^A^	346^AB^				
WSC	C	60.35^B^	60.24^B^	60.16^B^	56.89^C^	57.46^B^	56.37^B^	52.88^A^	1.11	<0.001	<0.001	<0.001
(g/kg DM)	J	60.12^B^	59.24^B^	51.02^C^	62.51^A^	62.92^A^	59.91^A^	55.51^A^				
	LB	66.31^A^	64.45^A^	60.95^B^	60.69^A^	61.23^A^	59.42^A^	58.44^A^				
	LP	65.28^AB^	64.60^A^	64.03^A^	61.70^A^	58.19^B^	58.21^AB^	55.02^A^				
	LPB	63.64^AB^	63.03^A^	61.55^B^	59.05^AB^	61.45^A^	58.96^A^	59.51^A^				
Crude protein	C	177.09^AB^	178.80^A^	175.10^A^	175.78^A^	175.95^A^	175.07^A^	172.32^BC^	0.23	<0.001	<0.001	<0.001
(g/kg DM)	J	174.63^B^	174.49^C^	175.43^A^	175.04^A^	174.76^A^	173.78^B^	175.48^A^				
	LB	177.54^A^	176.47^B^	174.54^A^	175.35^A^	175.85^A^	175.00^A^	172.50^B^				
	LP	175.26^AB^	176.91^B^	173.74^A^	175.95^A^	174.69^A^	173.95^B^	172.04^BC^				
	LPB	176.69^AB^	176.33^B^	172.87^A^	176.52^A^	174.47^A^	173.74^B^	171.82^C^				
NDF	C	488.86^A^	491.92^A^	480.54^A^	474.90^A^	488.06^A^	440.55^AB^	438.09^A^	3.03	<0.001	<0.001	<0.001
(g/kg DM)	J	483.39^A^	463.54^B^	466.72^A^	468.79^A^	458.00^B^	430.13^B^	442.82^A^				
	LB	488.81^A^	466.69^B^	464.74^A^	465.29^A^	468.62^B^	448.80^A^	446.66^A^				
	LP	470.76^A^	478.87^AB^	465.34^A^	466.42^A^	468.08^B^	441.31^AB^	448.66^A^				
	LPB	472.91^A^	475.93^AB^	458.47^A^	467.82^A^	465.63^B^	456.28^A^	445.57^A^				
ADF	C	317.35^B^	341.06^A^	338.47^A^	315.28^B^	325.03^A^	316.01^B^	322.03^A^	1.60	0.025	<0.001	<0.001
(g/kg DM)	J	348.93^A^	315.36^B^	337.72^A^	327.71^AB^	321.47^A^	312.19^B^	334.51^A^				
	LB	311.25^B^	324.56^AB^	316.33^B^	337.24^A^	317.88^A^	324.04^B^	327.26^A^				
	LP	303.63^B^	319.57^AB^	316.69^B^	328.13^AB^	329.51^A^	314.64^B^	324.42^A^				
	LPB	325.29^B^	337.80^AB^	312.50^B^	344.93^A^	335.82^A^	350.29^A^	324.61^A^				
Hemicellulose	C	171.51^A^	150.86^A^	142.07^A^	159.62^A^	163.03^A^	124.54^A^	116.07^AB^	2.72	<0.001	<0.001	<0.001
(g/kg DM)	J	134.46^A^	148.18^A^	129.00^A^	141.08^AB^	136.54^A^	117.94^AB^	108.31^B^				
	LB	177.56^A^	142.13^A^	148.40^A^	128.05^AB^	150.74^A^	124.76^A^	119.40^AB^				
	LP	167.12^A^	159.30^A^	148.65^A^	138.30^AB^	138.57^A^	126.67^A^	124.24^A^				
	LPB	147.62^A^	138.13^A^	145.97^A^	122.89^B^	129.82^A^	105.99^B^	120.96^AB^				

*^A–C^Within a day of ensiling, groups without a common superscript differed (P < 0.05).*

*^1^DM, dry matter; FW, fresh weight; WSC, water-soluble carbohydrates; NDF, neutral detergent fiber; ADF, acid detergent fiber.*

*^2^C, control; J, pre-fermented juice-treated silage; LP, L. plantarum-treated silage; LB, L. buchneri-treated silage; LPB, L. plantarum + L. buchneri-treated silage.*

*^3^SEM, standard error of mean.*

*^4^T, treatments; D, ensiling days; T × D, interaction between treatments and ensiling days.*

### Microbial Community

**FIGURE 1:**
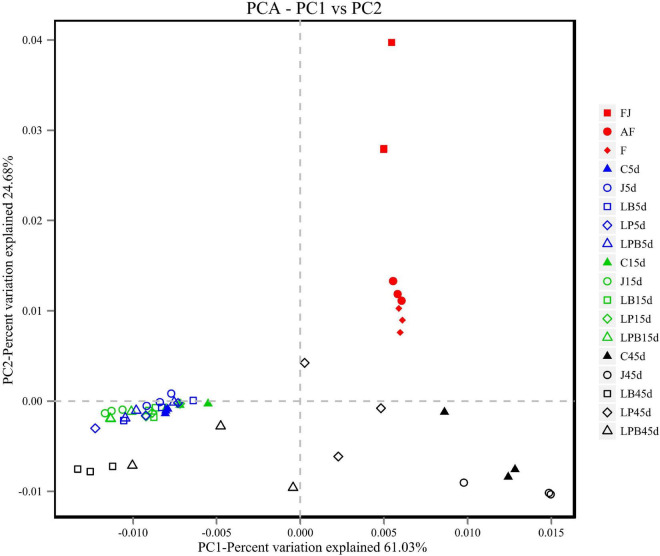
Principal coordinate analysis of the bacterial community in mixed silage. AF, fresh lucerne; RS, rice straw; WB; wheat bran; F, mixture; C, control; J, pre-fermented juice-treated silage; LP, *L. plantarum*-treated silage; LB, *L. buchneri* treated silage; LPB, *L. plantarum* + *L. buchneri*-treated silage.

**FIGURE 2:**
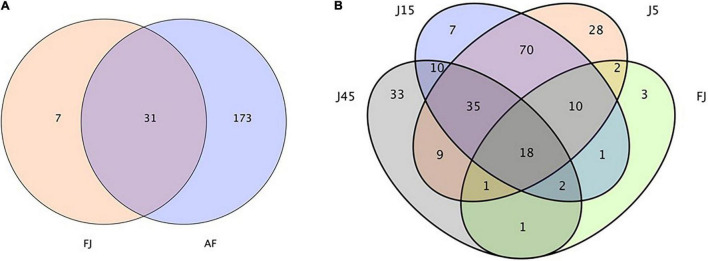
**(A)** Diagram of the operational taxonomic units between the AF, fresh lucerne and FJ, pre-fermented juice. **(B)** Venn analysis for operational taxonomic units FJ and J, pre-fermented juice-treated silage.

A total of 514,997 quality sequencing reads were generated by SMRT sequencing of the 16S rRNA gene (full-length) in 9 raw materials and 45 silages samples; based on 3% dissimilarity, there were 306 operational taxonomic units (OTUs). The Simpson, Shannon index, and Chao1 values in raw materials were greater than (*P* < 0.05) those of silage samples after 45 days of ensiling (Table 4). Inoculants decreased the Simpson, Shannon index, and Chao1 values relative to the C group, during 5 days of ensiling. However, at 5 and 15 days of ensiling, Simpson, Shannon index, and Chao1 were lowest in J silage, whereas after 45 days of ensiling, these indices were lowest in J, LB, and LPB. In principal component analysis (PCA; Figure 1), at 5 and 15 days of ensiling, all groups were clustered in the second and third quadrants, whereas after 45 days of ensiling, LP and LPB were in the third quadrant and the remainder were assigned to the fourth quadrant. Based on Venn analysis, overlapping OTUs (31) between fresh alfalfa and pre-fermented juice accounted for 81.6% of pre-fermented juice OTUs (Figure 2A). In addition, there were 3 specific OTUs in pre-fermented juice, whereas 31 bacterial OTUs were shared by pre-fermented juice and J silage at 5 and 15 days of ensiling. Furthermore, after 45 days of ensiling, fewer bacterial OTUs were shared by pre-fermented juice and J silage (Figure 2B).

**TABLE 4:** General information of sequence and bacterial diversity.

Day	Treatment	Item				
		Sequences	Shannon	Simpson	Chao 1	Good’s coverage
	FJ	25,200	0.31^G^	0.06^H^	36.53^E^	0.9986
	AF	28,782	5.30^A^	0.94^AB^	217.07^A^	0.9982
	F	27,594	5.82^A^	0.97^A^	207.13^AB^	0.9980
5 days	C	29,046	4.01^BCD^	0.85^ABCD^	190.93^AB^	0.9957
	J	24,185	3.67^BCDE^	0.83^ABCD^	155.11^ABC^	0.9958
	LB	26,077	3.87^BCD^	0.84^ABCD^	186.29^AB^	0.9953
	LP	29,194	3.84^BCD^	0.84^ABCD^	177.71^AB^	0.9966
	LPB	24,766	3.35^CDE^	0.80^BCD^	179.66^AB^	0.9956
15 days	C	29,056	4.30^B^	0.89^ABC^	186.63^AB^	0.9963
	J	28,633	3.13^DE^	0.72^CDE^	145.29^BC^	0.9974
	LB	27,288	3.83^BCD^	0.83^ABCD^	181.41^AB^	0.9964
	LP	28,675	4.10^BC^	0.86^ABC^	181.66^AB^	0.9963
	LPB	28,658	3.58^BCDE^	0.82^ABCD^	178.27^AB^	0.9951
45 days	C	31,814	2.85^E^	0.69^DEF^	98.04^CDE^	0.9991
	J	33,682	1.66^F^	0.56^EFG^	63.44^DE^	0.9992
	LB	32,309	1.45^F^	0.49^G^	50.23^DE^	0.9983
	LP	29,640	3.21^CDE^	0.80^ABCD^	104.41^CD^	0.9973
	LPB	30,398	1.73^F^	0.55^FG^	75.25^DE^	0.9988

*^A–H^Within a column, means without a common superscript differed (P < 0.05). AF, fresh lucerne; RS, rice straw; WB, wheat bran; F, mixture; C, control; FJ, pre-fermented juice; J, pre-fermented juice-treated silage; LP, L. plantarum-treated silage; LB, L. buchneri-treated silage; LPB, L. plantarum + L. buchneri-treated silage.*

**FIGURE 3:**
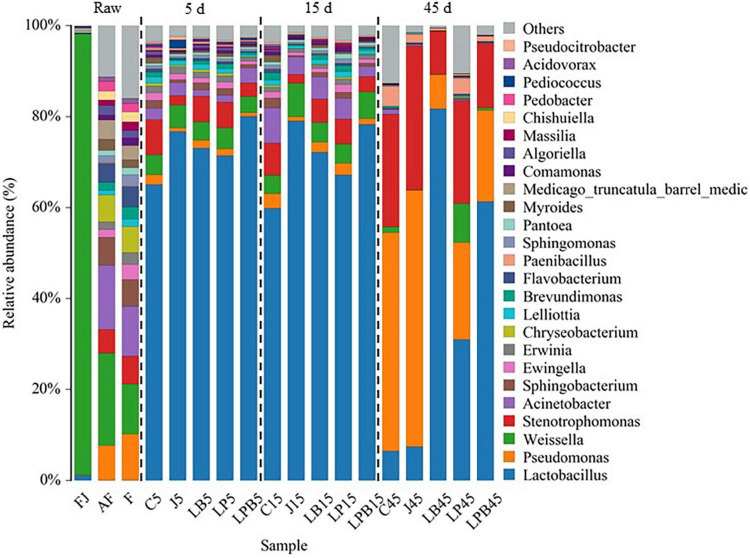
Bacterial community (genus level) of mixed silage. AF, fresh lucerne; FJ, pre-fermented juice; F, mixture; C, control; J, pre-fermented juice-treated silage; LP, *L. plantarum*-treated silage; LB, *L. buchneri*-treated silage; LPB, *L. plantarum* + *L. buchneri*-treated silage.

**FIGURE 4:**
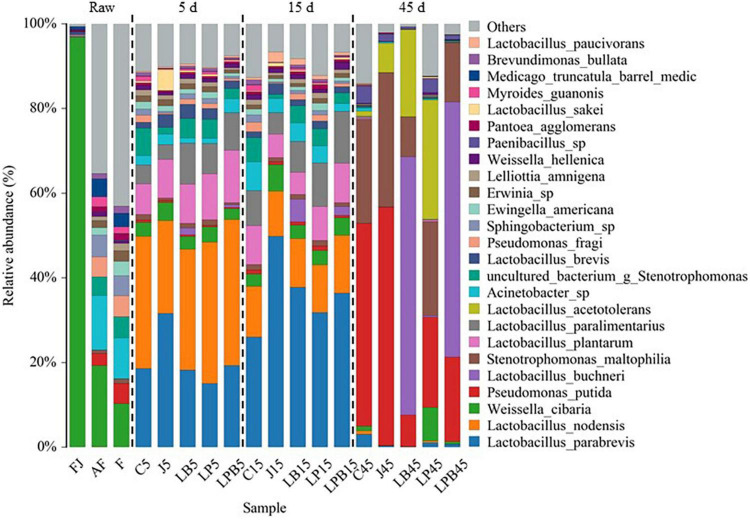
Bacterial community (species level) of mixed silage. AF, fresh lucerne; FJ, pre-fermented juice; F, mixture; C, control; J, pre-fermented juice-treated silage; LP, *L. plantarum*-treated silage; LB, *L. buchneri*-treated silage; LPB, *L. plantarum L. buchneri*-treated silage.

Relative abundances of bacteria (genus and species levels) are shown in Figures 3, 4, respectively. *Weissella*, *Acinetobacter*, and *Pseudomonas* were the main epiphytic bacteria of lucerne and mixture (Figure 3). Furthermore, *W. cibaria* (19.3 and 10.3%), *Acinetobacter* sp. (12.8 and 9.7%), *Sphingobacterium* sp. (5.2 and 4.8%), uncultured bacterium *Stenotrophomonas* (4.4 and 5%), *Pseudomonas putida* (2.8 and 4.7%), and *Pseudomonas fragi* (4.8 and 4.9%) were the major bacterial species in lucerne and mixture (Figure 4). In pre-fermented juice, there were six predominant genera: *Weissella* (97%), *Lactobacillus* (1.1%), *Pediococcus* (0.4%), *Pantoea* (0.4%), *Pseudomonas* (0.1%), and *Sphingomonas* (0.1%). The major microbiota species in pre-fermented juice were *W. cibaria* (96.9%), *L. brevis* (0.6%), *Pantoea agglomerans* (0.4%), *L. plantarum* (0.3%), *L. paralimentarius* (0.3%), and *Pseudomonas putida* (0.1%). After 5 and 15 days of ensiling, LA-producing bacteria (*Lactobacillus*, *Weissella*, and *Pediococcus*) were the dominant genera (64.1–84.3%) in all silage samples. Furthermore, for all groups, at 5 and 15 days of ensiling, dominant LAB species were *L. parabrevis*, *L. nodensis*, *W. cibaria*, *L. buchneri*, *L. plantarum*, *L. paralimentarius*, *L. brevis*, *W. hellenica*, *L. sakei*, and *L. paucivorans*. However, after 45 days of ensiling, *Pseudomonas putida* and *Stenotrophomonas maltophilia* sharply increased and remained predominant, followed by *L. buchneri* and *L. acetotolerans*.

**FIGURE 5:**
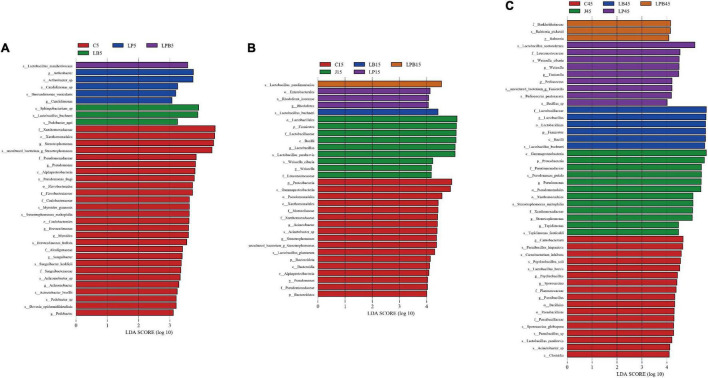
Comparison of microbial variations during the ensiling using the Latent Dirichlet allocation effect size (LEfSe) analysis using the Kruskal-Wallis test (*P* < 0.05) with linear discriminant analysis (LDA) score > 3.0. 5 days **(A)**, 15 days **(B)**, and 45 days **(C)** of ensiling. C, control; J, pre-fermented juice-treated silage; LP, *L. plantarum*-treated silage; LB, *L. buchneri*-treated silage; LPB, *L. plantarum* + *L. buchneri*-treated silage.

**FIGURE 6:**
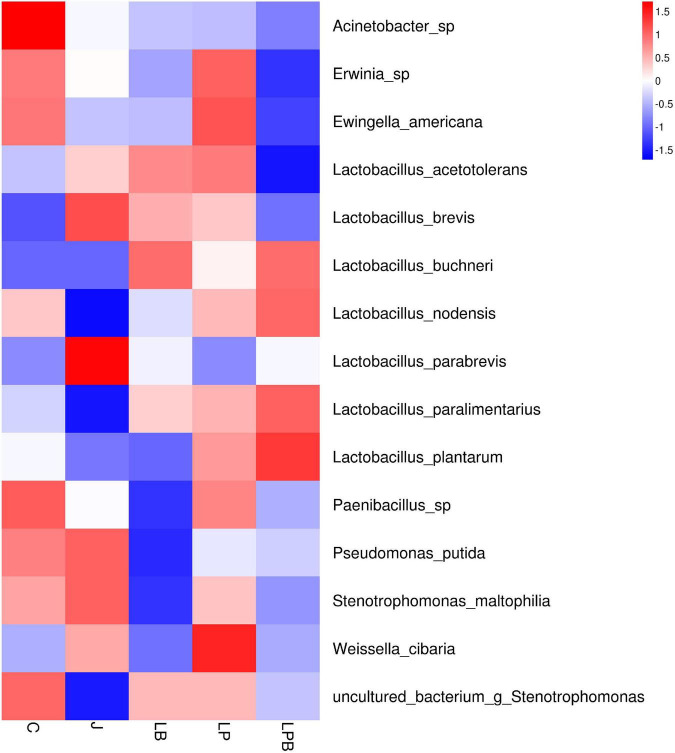
Heatmap of prominent bacterial species (15 most abundant genera) for alfalfa mixed silage. C, control; J, pre-fermented juice-treated silage; LP, *L. plantarum*-treated silage; LB, *L. buchneri*-treated silage; LPB, *L. plantarum L. buchneri*-treated silage.

Differences in microbial communities of silages during ensiling are shown (Figures 3–6). Relative abundances of LA-producing bacteria at the genus level in inoculant treatments were higher than C silage (69.8 vs. 64%), whereas there were greater abundances of LA-producing bacteria in J (81.9 and 86.4%) and LPB (83.6 and 84.3%) silages than that of other treatments at 5 and 15 days of ensiling (Figure 3). After 5 days of ensiling, except for J silage, *L. nodensis* was the most abundant species, whereas *L. parabrevis* remained predominant in J silage, followed by *L. nodensis.* Furthermore, compared to other treatments, J silage had more *W. cibaria* and *L. sakei* after 5 days of ensiling. *L. parabrevis* gradually replaced *L. nodensis* as the dominant species in all silage samples after 15 days of ensiling, whereas compared to C silage, relative abundance of *L. parabrevis* was increased by 23.8, 11.7, 5.8, and 10.3% in J, LB, LP, and LPB silages, respectively. For the J silage, there were more *W. cibaria*, whereas *L. paralimentarius* had the highest abundance in LPB silage after 15 days of ensiling. However, *Acinetobacter lwoffii* and *Acinetobacter* sp. were observed in C silage at Days 5 and 15 of ensiling, respectively, and Enterobacterales was identified in LP silage after 15 days of ensiling (Figures 5A,B).

Overall, during 45 days of ensiling, the dominant species in C and J silages were *Pseudomonas putida* and *Stenotrophomonas maltophilia*, whereas in LP and LPB, *L. buchneri* was the most abundant. *L. acetotolerans* was the dominant species in LP and was also present in J and LB after 45 days of ensiling. There was greater relative abundance of *Psychrobacillus soli*, *Sporosarcina globispora*, *Carnobacterium inhibents*, *Paenibacillus hispanicus*, and *Acinetobacter* sp. in C silage compared to other treatments during 45 days of ensiling (Figure 5C). Heatmaps (species level) of main bacterial communities in mixed silage are shown (Figure 6). There was a positive correlation with C silage and *Acinetobacter* sp., *Erwinia* sp., *Ewingella americana*, *Paenibacillus* sp., and uncultured *Stenotrophomonas*. In addition, there were positive correlations between J silage and *L. parabrevis*, *L. brevis*, *Pseudomonas putida*, and *Stenotrophomonas maltophilia*, whereas there were negative correlations between LB silage and *Pseudomonas putida*, *Stenotrophomonas maltophilia*, and *Paenibacillus* sp., whereas LP silage was positively correlated with *W. cibaria*, *Erwinia* sp., *Ewingella americana*, *Paenibacillus* sp., and *L. acetotolerans*. In addition, LPB silage was positively correlated with *L. plantarum*, *L. paralimentarius*, *L. buchneri*, and *L. nodensis*, but had negative correlations with *Erwinia* sp., *Ewingella americana*, and *L. acetotolerans*.

The succession of inoculations in the silage environment is shown in Table 5. As *W. cibaria* was dominant in pre-fermented juice, we focused on *W. cibaria* succession in J silage. The addition of pre-fermented juice tended to cause greater abundances of *W. cibaria* in J vs. C silages after 5 days of ensiling, and higher (*P* < 0.01) relative abundances of *W. cibaria* (relative to other inoculated silages) after 15 days of ensiling. Furthermore, inoculation with *L. plantarum* had no obvious effect on increasing relative abundance of *L. plantarum* at 5, 15, or 45 days of ensiling. Relative abundance of *L. buchneri* in silages not inoculated with this bacterium was very limited (<1%) over the entire ensiling period, whereas inoculation with *L. buchneri* strains increased its relative abundance, with a sharp increase in LB and LPB silages after 45 days of ensiling.

**TABLE 5:** The succession of inoculations in a silage environment.

Day	Treatment	*Weissella cibaria* (%)	*L. plantarum* (%)	*L. buchneri* (%)
5 days	C	3.24^A^	7.31^A^	<0.01^B^
	J	4.35^A^	9.03^A^	<0.01^B^
	LB	3.07^A^	9.14^A^	1.59^A^
	LP	3.68^A^	10.78^A^	0.00^B^
	LPB	2.57^A^	12.23^A^	0.56^B^
SEM		0.23	0.77	0.17
15 days	C	2.95^B^	9.36^A^	<0.01^C^
	J	6.17^A^	5.50^A^	<0.01^C^
	LB	3.24^B^	5.17^A^	5.33^A^
	LP	3.41^B^	8.05^A^	<0.01^C^
	LPB	4.16^AB^	9.28^A^	2.13^B^
SEM		0.36	0.61	0.56
45 days	C	1.09^A^	0.32^A^	0.01^B^
	J	0.09^A^	0.04^A^	0.01^B^
	LB	0.05^A^	0.01^A^	61.00^A^
	LP	7.30^A^	0.36^A^	0.31^B^
	LPB	0.54^A^	0.07^A^	60.64^A^
SEM		1.28	0.06	8.38

*^A–C^Within a column and sampling day, means without a common superscript differed (P < 0.05).*

*AF, fresh lucerne; RS, rice straw; WB; wheat bran; F, mixture; C, control; J, pre-fermented juice-treated silage; LP, L. plantarum-treated silage; LB, L. buchneri-treated silage; LPB, L. plantarum + L. buchneri-treated silage.*

**FIGURE 7:**
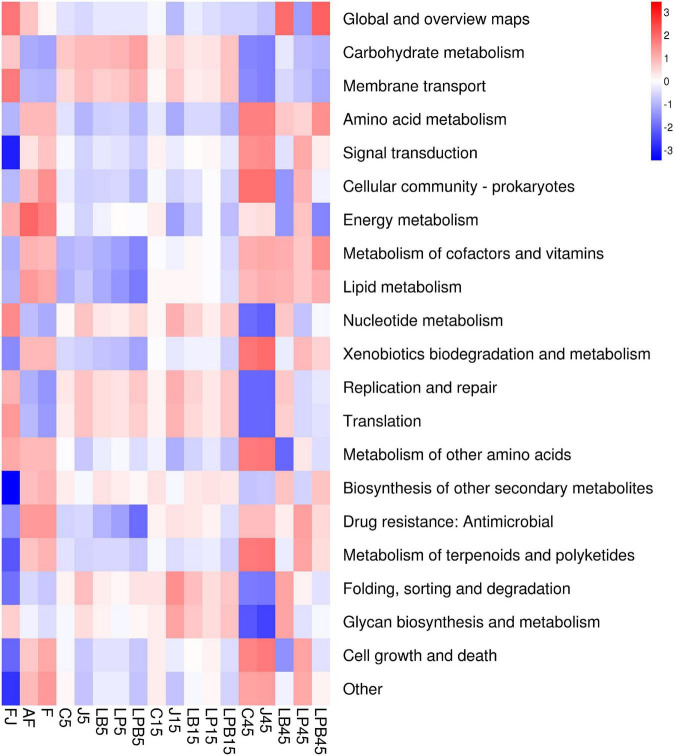
Heatmap of KEGG pathway, the second level of the predicted functional shift is shown with respect to the fermentation processes and inoculant treatments. AF, fresh alfalfa; FJ, pre-fermented juice; F, mixture; C, control; J, pre-fermented juice-treated silage; LP, *L. plantarum*-treated silage; LB, *L. buchneri*- treated silage; LPB, *L. plantarum* + *L. buchneri*-treated silage.

A heatmap of KEGG metabolic pathways during the ensiling process is shown in Figure 7. Metabolic pathways changed with ensiling days, with carbohydrate metabolism and cofactors and vitamins positively correlated with silage samples after 5 and 15 days, but negatively correlated with silage samples after 45 days. However, silage samples were negatively correlated with amino acid metabolism and nucleotides after 5 and 15 days, whereas the opposite trend was observed after 45 days of ensiling. *L. buchneri* inoculants increased abundances of global and overview maps compared to other treatments.

### Aerobic Stability

**FIGURE 8:**
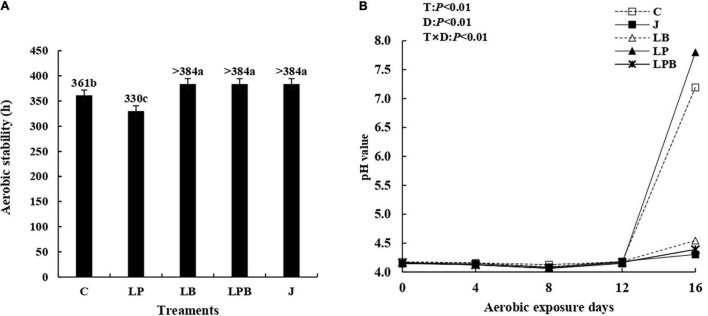
Aerobic stability **(A)** and dynamics of pH **(B)** during aerobic exposure days. C, control; J, pre-fermented juice-treated silage; LP, *L. plantarum*-treated silage; LB, *L. buchneri*-treated silage; LPB, *L. plantarum* + *L. buchneri*-treated silage. D, ensilage days; T, treatments; T × D, the interaction between treatment and ensiling days.

The aerobic stability of silage treated with *L. buchneri* and pre-fermented juice improved significantly, relative to C and LP silage, with LB, LPB, and J silages stable for > 384 h. The pH of aerobic stability was affected (*P* < 0.01) by inoculant, ensiling days, and their interaction (Figures 8A,B). For C and LP silages, there was a sharp rise in pH after 12 days of aerobic exposure.

## Discussion

### Characteristics of Fresh Material and Pre-fermented Juice

The LAB count and pH in pre-fermented juice before ensiling were 7.61 log cfu/ml and 4.16, consistent with the previous studies (Sun et al., 2021). When 80% lucerne was mixed with RS and WB, the adjusted DM content was > 360 g/kg, which met requirements for ideal DM (range of 300–400 g/kg for quality silage; McDonald et al., 1991).

### Effect of Pre-fermented Juice and Inoculants Ensiling on Silage Quality

Adding LAB inoculants could accelerate the decline of silage pH by enhancing LA- fermentation and inhibiting undesirable bacteria (Adesogan, 2008). In the study, we also observed higher LA concentration, lower pH and AN concentration at earlier stages of ensiling in all inoculants treated silages than C silage. Applying a combination of homofermentative LAB and heterofermentative LAB captured the benefits of both types of bacteria while overcoming their drawbacks (Adesogan, 2008). In this study, LPB silage had the lowest pH over the ensiling period. Similarly, there was rapid and substantial LA production and the initial rate of acidification was also increased in LP and LB-inoculated low-DM corn and sorghum silages (Filya, 2003). In the current study, inoculation with pre-fermented juice increased LA, AA, and PA concentrations and decreased pH rapidly after 3 days of ensiling. Wang et al. (2009) reported that addition of pre-fermented juice accelerated the early decline of pH to a lower level than control alfalfa silage, due to faster accumulation of LA at the start of ensiling. The number of bacterial OTUs shared by pre-fermented juice and J silage was higher at 5 or 15 days than at 45 days. Addition of pre-fermented juice had more obvious effects on microorganisms in the early stages of ensiling. Furthermore, inoculation with pre-fermented juice decreased silage AN concentration over the entire ensiling interval. Similar to that of Sun et al. (2021), who added pre-fermented juice to lucerne silage and found that pre-fermented juice efficacity decreased pH and AN and increased LA content. The possible reason was as follows: first, exogenous microbiota prepared from epiphytic LAB of lucerne were more likely to adapt and reconstitute in lucerne compared to LAB derived from other crops during ensiling; second, inoculation with pre-fermented juice could stimulate LAB to use fermentable substances at the onset of ensiling (Ohshima, 1997; Shao et al., 2007). In addition, Ali et al. (2020) reported extracted epiphytic microbiota from red clover inoculation more rapidly decreased pH in sterile red clover at earlier stages of ensiling than extracted epiphytic microbiota from maize and sorghum. In addition, higher AA concentration for pre-fermented juice treatments suggested some heterofermentative pathways were promoted during ensiling (Ohshima, 1997). In addition, these results supported the notion that inoculation of lucerne with pre-fermented juice derived from barley, wheat, and grass herbages increased silage AA after 45 days of ensiling (Denek et al., 2011), as our results had a similar tendency. In the current study, concentrations of LA and AA increased most slowly during the preliminary stages of ensiling with *L. buchneri* inoculants, whereas AA concentrations were higher in LB vs. C silage after 45 days of ensiling. *L. buchneri* usually became active after 45 days of ensiling (Muck, 2010). Inoculating with LB resulted in the greatest AN after 45 days of ensiling, perhaps due to the slower LA, AA production, and pH decline in LB silage during 5 days of ensiling. This agrees with previous reports that increased AN with *L. buchneri* inoculants in corn silage was associated with the increased potential for pH during the ensiling (Driehuis et al., 2001). Others also observed increased AN concentration in corn and sorghum silages treated with LB (Filya, 2003).

### Effect of Pre-fermented Juice and Inoculants on Silage Chemical Composition

Lower residual WSC content of silage indicated higher DM losses during ensiling, producing worse silage quality (Weinberg et al., 1993). That the C silage had less DM and WSC after 7 days of ensiling was attributed to proliferation of undesirable bacteria increasing nutrient losses. In addition, the WSC content of J silage decreased more rapidly compared to that of other treatments during 5 days of ensiling, perhaps due to this silage having more LA and AA. Similarly, lower WSC in calcium propionate-treated silage after 45 days of ensiling was attributed to higher LA and AA (Mu et al., 2021). Concentration of CP was higher in J silage than C silage on Day 45. This agrees with previous reports of higher CP content in pre-fermented juice-treated silage than un-treated silage in total mixed ration silage (Yanti et al., 2019). In addition, pre-fermented juice was better than LAB for inhibiting proteolysis, based on non-protein N concentrations in alfalfa silage (Wang et al., 2009). The contents of NDF and HC decreased markedly among all silages at 45 days of ensiling, whereas J silage had the lowest HC content at 45 days of ensiling. This may be related to the structural carbohydrates, which could be hydrolysis by organic acids after a long fermentation (Wang et al., 2018). The decline of NDF and HC contents during ensiling of corn silage could possibly be due to the acid hydrolysis of the cell wall fraction (Huisden et al., 2009). Hence, greater concentration of LA and AA in J silage could explain the lowest HC in J silage after 45 days of ensiling. However, there were no significant differences in NDF and ADF contents in inoculant treatments vs. C silage after 45 days of ensiling. This is similar to that of Wang et al. (2009), who reported that applying pre-fermented juice as an additive did not affect NDF and ADF contents of alfalfa silage. This contradicts Denek et al. (2012) who reported that inoculation contributed to lower NDF and ADF contents in alfalfa silage because some bacteria are able to breakdown cellulose and HC.

### Effect of Pre-fermented Juice and Inoculants Ensiling on Silage Bacterial Community

Greater bacterial diversity and richness in raw materials than those of silage were due to the unviability of some epiphytic microbes under the aerobic and acerbic environment. Yuan et al. (2019) reported that the bacterial diversity index was greater in fresh materials than Napier grass silages. The addition of inoculants decreased the Simpson, Shannon index, and Chao1 values as compared to C silage after 5 days of ensiling, whereas J silage had the lowest bacterial diversity and richness after Day 5 of ensiling, attributed to the sharp initial decline in pH. Inoculating LAB could decrease silage pH, which inhibited the growth of undesirable microbes with lower microbial diversity compared to untreated alfalfa silage (Yang et al., 2019). After 45 days of ensiling, the higher Simpson, Shannon index, and Chao1 values in C and LP silages vs. other groups may have been due to more undesirable microbes (Figure 6).

Tao et al. (2018) observed the *Lactobacillus* and *Pediococcus* in pre-fermented juice prepared from lucerne using agar media-based culturing method, while seven common LAB species were identified in lucerne pre-fermented juice using 16S rRNA gene sequencing techniques (Sun et al., 2021). However, in this study four species (*W. cibaria*, *L. brevis*, *L. plantarum*, and *L. paralimentarius*) of LAB in three genera were present in pre-fermented juice, with *W. cibaria* (96.9%) being dominant. Sun et al. (2021) also found that *W. cibaria* was identified in the pre-fermented juice prepared from lucerne. Preparation of pre-fermented juice involved culturing microbes on the crop surface. That 81.6% of microorganisms in pre-fermented juice were also present in fresh lucerne confirmed that epiphytic microorganisms affected the composition of the microbial community in pre-fermented juice. Furthermore, *Weissella*, *Lactobacilli*, and *Cocci* species were reported as major components in various crops (Cai et al., 1998).

The addition of exogenous microbial inoculant enriched LA-producing bacteria after 5 and 15 days of ensiling, whereas adding LP enriched *Lactobacillus* and *Weissella* of cauliflower leaf silages at 30 days of ensiling (Ren et al., 2020). There were greater abundances of *Lactobacillus*, *Weissella*, and *Pediococcus* in J and LPB silages at Days 5 and 15 of ensiling, which may have promoted rapid and efficient LA fermentation and faster pH reduction early in ensiling. Similarly, silages treated with pre-fermented juice had higher LAB counts and LA contents, but lower pH than untreated lucerne silage (Sun et al., 2021). In the current study, *L. nodensis* and *L. parabrevis* were the predominant bacteria in all groups on Days 5 and 15 of ensiling. Kashiwagi et al. (2009) isolated *L. nodensis* from rice bran; however, there is limited information about this species in silage. Perhaps the presence of *L. nodensis* was associated with a rapid increase in LA and decline in pH during 5 days of ensiling. Pre-fermented juice has the potential to promote the shift of *L. nodensis* to *L. parabrevis* during15 days of ensiling, and *L. parabrevis* had a greater abundance in J silage than other treatments at Days 5 and 15 of ensiling. Xu et al. (2021) observed that *L. parabrevis* was enriched in all treatments of whole crop corn silage after 7 days of ensiling. Wu et al. (2020) reported that LP-treated silage increased the abundance of *L. parabrevis* in high-moisture sweet corn kernel silage. In addition, more *W. cibaria* and *L. sakei* were observed in J silage than other treatments after 5 and 15 days of ensiling. Bai C. et al. (2021) speculated that *W. cibaria* maintained a high relative abundance in alfalfa silage because of their stronger vitality and competitiveness. That *W. cibaria* was the dominant species in pre-fermented juice accounted for its competitiveness in J silage. *L. sakei* had a significant role in fermentation of grass, with concentrations of LA and AA positively correlated with this bacterium (Ding et al., 2020). In the present study, the LPB silage had the highest abundance of *L. paralimentarius* after 15 days of ensiling. Similarly, inoculation of ensiled corn with LB and LP increased relative abundances of *L. paralimentarius* (Xu et al., 2021). After 45 days of ensiling, *Pseudomonas putida* and *Stenotrophomonas maltophilia* in C and J silages reached the highest relative abundance. Some species of *Pseudomonas* are usually associated with decreases in AN concentration and yeast count, and they also have been reported in other silages such as mulberry leaf and sweet sorghum (Wang et al., 2019; Ren et al., 2021). In the current study, J silage tended to have lower AN concentration, attributed to a greater abundance of *Pseudomonas putida*. Dong et al. (2020) assessed the microbial communities of ryegrass silage and reported a positive correlation between *Stenotrophomonas* and LA concentrations. *Stenotrophomonas maltophilia* was also isolated from fermented high-moisture sweet corn kernel (Wu et al., 2020). In this study, there was a high abundance of spoilage-producing organisms, including *Acinetobacter* sp., *Erwinia* sp., *Paenibacillus* sp., *Paenibacillus hispanicus*, *Psychrobacillus soli*, and *Carnobacterium inhibents* in C silage, and Enterobacterales, *Paenibacillus* sp., and *Erwinia* sp., in LP silage. However, inoculating with *L. buchneri* inoculants could inhibit *Erwinia* sp. and *Paenibacillus* sp. Considerable abundance of *Acinetobacter* was detected in *Neolamarckia cadamba* leaf, which can use AA as substrates and decrease silage quality under anaerobic conditions (He et al., 2020). A lower relative abundance of *Erwinia* in total mixed ration silage was associated with the improved silage quality (Jiang et al., 2020), as this genus belongs to *Enterobacteria*, which can compete with LAB for substrate. Both *Paenibacillus* and *Psychrobacillus* may promote growth of less acid-tolerant spoilage microorganisms; they were detected in Napier grass silage and mixed corn and soybean silage (Zeng et al., 2020; Yin et al., 2021). Furthermore, *Carnobacterium* apparently decreased fermentation quality of Italian ryegrass silage (Yan et al., 2019).

### The Succession of Three Inoculants Over Ensiling

In most studies, regardless of whether *Weissella* was present in fresh forage or silage, eventually, *Lactobacilli* became dominant during terminal fermentation (Cai et al., 1998; Mu et al., 2021). *W. cibaria* initiated lactate fermentation in silage, creating an aerobic environment suitable for development of *Lactobacilli*, although they grew vigorously only during the early stage of ensiling. Inoculants may have undesirable effects due to a lack of compatibility between the additive and plant material (Koc et al., 2017). In this study, adding *L. plantarum* treatments did not obviously increase the relative abundance of this bacterium, with more spoilage-inducing organisms present in LP silage. In previous studies with *L. buchneri*, a longer conservation period (from 45 to 90 days) was needed to be efficacious (Arriola et al., 2021b). Likewise, in this study, a dramatic shift in microbial composition occurred in LB and LPB silages after 45 days of ensiling, with massive amounts of *L. buchneri* as assessed with SMRT.

### Effect of Kyoto Encyclopedia of Genes and Genomes Metabolism Pathways in Raw Material and Silages

Functional predictions of bacterial communities facilitate assessment of effects of bacterial communities on changes in metabolic pathways underlying silage formation (Bai J. et al., 2021). In the present study, the metabolic pathways changed with time. There was a high incidence of carbohydrate metabolism in silages from 5 to 15 days of ensiling, whereas metabolism of cofactors and vitamins, and amino acid metabolism had a high incidence only after 45 days of ensiling. Expression of the carbohydrate metabolism pathway may be related to active LAB metabolism during early ensiling, with accumulation of much LA and VFA during 15 days of ensiling. Similarly, Bai J. et al. (2021) reported higher relative abundances of carbohydrate metabolism during the early stage of ensiling alfalfa treated with *L. plantarum* and *P. pentosaceus*. Higher amino-acid metabolism at the end of ensiling was caused by undesirable microbes that increased AN concentration at 45 days of ensiling; conversely, high amino-acid metabolism may be an attempt to degrade macromolecular proteins into readily absorbable amino acids or peptides (Bai J. et al., 2021; Du et al., 2021b). In addition, *L. buchneri* inoculants promoted global metabolism, perhaps due to this bacterium suppressing harmful microorganisms in LB and LPB silages. Du et al. (2021a) also reported that superior global metabolism in woody plant mixed silage was due to inhibition of harmful microorganisms by extensive LA-fermentation.

### Effect of Inoculants Ensiling on Aerobic Stability

Aerobic stability was improved with LB-treated silages, which is not only due to more AA accumulation after 45 days of ensiling but also some antimicrobial substances like bacteriocin produced by *L. buchneri* (Arriola et al., 2021a). The recent meta-analysis also reported that *L. buchneri*-based inoculants improved aerobic stability by increased AA concentration and reduced yeast counts (Arriola et al., 2021a). According to Tao et al. (2017), the pre-fermented juice application prolonged aerobic stability by 17 h in comparison to the control. Similarly, in this study, pre-fermented juice significantly improved the aerobic stability of silage, which is likely due to the increased relative abundance of *L. brevis*. *L. brevis* is known as a hetero-fermentative strain belonging to the *L. buchneri* group of lactobacilli, which showed antibacterial and probiotic properties (Soundharrajan et al., 2019).

## Conclusion

In this study, four species of LAB from three genera were detected in pre-fermented juice, with *W. cibaria* dominating. Adding LPB increased abundance of *L. plantarum*, *L. paralimentarius*, and *L. nodensis* and resulted in the lowest pH. Pre-fermented juice enriched abundance of *W. cibaria*, *L. sakei*, *L. parabrevis*, *Pseudomonas putida*, and *Stenotrophomonas maltophilia*, enhanced accumulation of AA and LA, rapidly decreased pH, and reduced CP losses, AN, and HC. Aerobic stability was prolonged (>384 h) by inoculating with LB, LPB, or pre-fermented juice; the latter is a promising biological source of LAB for cleaner production to improve silage quality.

## Data Availability Statement

Publicly available datasets were analyzed in this study. This data can be found here: The sequencing data were submitted to the NCBI Sequence Read Archive database (accession number: PRJNA798538). https://www.ncbi.nlm.nih.gov/bioproject/PRJNA798538.

## Author Contributions

LM: conceptualization, methodology, and writing—review and editing. QW: resources and formal analysis. XC: investigation and data curation. HL: methodology and investigation. ZZ: validation and supervision. All authors contributed to the article and approved the submitted version.

## Conflict of Interest

The authors declare that the research was conducted in the absence of any commercial or financial relationships that could be construed as a potential conflict of interest.

## Publisher’s Note

All claims expressed in this article are solely those of the authors and do not necessarily represent those of their affiliated organizations, or those of the publisher, the editors and the reviewers. Any product that may be evaluated in this article, or claim that may be made by its manufacturer, is not guaranteed or endorsed by the publisher.

## Footnotes

1
http://www.biocloud.net/
